# In-stent restenosis in acute coronary syndrome—a classic and a machine learning approach

**DOI:** 10.3389/fcvm.2023.1270986

**Published:** 2023-12-22

**Authors:** Alexandru Scafa-Udriște, Lucian Itu, Andrei Puiu, Andreea Stoian, Horatiu Moldovan, Nicoleta-Monica Popa-Fotea

**Affiliations:** ^1^ Department of Cardio-Thoracic Pathology, University of Medicine and Pharmacy “Carol Davila”, Bucharest, Romania; ^2^ Department of Cardiology, Emergency Clinical Hospital, Bucharest, Romania; ^3^ Department of Image Fusion and Analytics, Siemens SRL, Brasov, Romania; ^4^ Automation and Information Technology, Transilvania University of Brasov, Brasov, Romania

**Keywords:** risk factors, prediction, machine learning algorithms, in-stent restenosis, acute coronary syndrom(s)

## Abstract

**Background:**

In acute coronary syndrome (ACS), a number of previous studies tried to identify the risk factors that are most likely to influence the rate of in-stent restenosis (ISR), but the contribution of these factors to ISR is not clearly defined. Thus, the need for a better way of identifying the independent predictors of ISR, which comes in the form of Machine Learning (ML).

**Objectives:**

The aim of this study is to evaluate the relationship between ISR and risk factors associated with ACS and to develop and validate a nomogram to predict the probability of ISR through the use of ML in patients undergoing percutaneous coronary intervention (PCI).

**Methods:**

Consecutive patients presenting with ACS who were successfully treated with PCI and who had an angiographic follow-up after at least 3 months were included in the study. ISR risk factors considered into the study were demographic, clinical and peri-procedural angiographic lesion risk factors. We explored four ML techniques (Random Forest (RF), support vector machines (SVM), simple linear logistic regression (LLR) and deep neural network (DNN)) to predict the risk of ISR. Overall, 21 features were selected as input variables for the ML algorithms, including continuous, categorical and binary variables.

**Results:**

The total cohort of subjects included 340 subjects, in which the incidence of ISR observed was 17.68% (*n* = 87). The most performant model in terms of ISR prediction out of the four explored was RF, with an area under the receiver operating characteristic (ROC) curve of 0.726. Across the predictors herein considered, only three predictors were statistically significant, precisely, the number of affected arteries (≥2), stent generation and diameter.

**Conclusion:**

ML models applied in patients after PCI can contribute to a better differentiation of the future risk of ISR.

## Introduction

Coronary artery disease (CAD), especially acute myocardial infarction (AMI), is one of the most common causes of death worldwide despite the developments in diagnosis and revascularization treatment (1, 2). In-stent restenosis (ISR), defined by lumen reduction following a percutaneous coronary intervention (PCI) by more than 50% or at least 5 mm in a stent edge (3), is determined by one of the two processes: neo-atherosclerosis, defined by the accumulation of lipids and macrophages within the neointima, with or without necrotic core and calcifications, and neointimal proliferation, caused by the proliferation and migration of vascular smooth muscle cells into the tunica intima layer (4). In-stent restenosis is a progressive process that might begin early (within days) from the PCI or late (weeks to months) and it correlates with three major underlying mechanisms: elastic recoil, vascular remodeling and neointimal hyperplasia (5). In-stent restenosis continues to pose a significant problem even in the age of drug-eluted stents (DES), although compared to bare-metal stents (BMS), DES proved to reduce restenosis and postpone later revascularization by a total percent of 50%–70% (3). The incidence rate of ISR in the case of DES, although lower than BMS remains as high as 10% (6). For this reason, ISR has been thoroughly studied throughout the years, in order to find out the best approach to prevent and treat restenosis. Unfortunately, the risk factors for restenosis remain controversial, only a few of them (diabetes mellitus, stent length and stent diameter) being consistently found to influence the rate of coronary ISR (7–9).

In some studies, classic cardiovascular risk factors are deemed to increase the likelihood of ISR (9, 10), but in some others this relationship is not very clearly validated (11). Many studies investigated the role of various cardiovascular risk factors in the development of restenosis; diabetes mellitus was incriminated in many researches as an independent risk factor for ISR, along with arterial hypertension, active smoking and low-density lipoprotein cholesterol (LDL-C) (12–14). Erkan Yıldırım et al. (15) showed in multivariate logistic regression that ISR was independently associated with Gensini score, stent diameter and length, left ventricle ejection fraction, and LDL-C. In another study concentrating on the risk of restenosis in patients with sirolimus-eluting stent implantation, age, arterial hypertension (HTN), diabetes mellitus (DM), LDL-C, high sensitivity C-reactive protein (hsCRP), and target lesion on left circumflex artery (LCX) were independent predictive factors for restenosis (12). Other reports concentrated on the angiographic risk factors of ISR; several studies (9, 12) as well as a meta-analysis (7) showed that increased stent length is correlated with ISR, while the stent diameter implication in restenosis is debatable with two studies pinpointing increase of ISR with reduced stent diameter (13, 15), while another study found the opposite, that ISR correlates with increased stent diameter (9). Multi-vessel disease or multiple stents implanted also increases the risk for ISR (14). The location of the implanted stent is also correlated with the risk of restenosis, left anterior descending artery (LAD) being the most often cited as the location with the highest risk of ISR (9), although there are many studies citing either LCX or right coronary artery (RCA) (7, 12). Inflammatory markers such as CRP or hsCRP also correlated with ISR (12, 14). Moreover, single nucleotide polymorphisms such as: fibrinogen factor I (rs1800790), monocyte differentiation antigen CD14 (rs2569190), and nitric oxide synthase 3 (rs1799983) have been correlated with ISR (16). Even if the precise value of these cardiovascular risk factors is not clear due to some contradictory findings, we have integrated some of these classic risk factors and completed with other parameters, related to the angiographic findings using a new approach based on machine learning (ML) that may produce a better differentiation of the future risk of ISR.

Recently, ML technology started to be used in medical studies due to its ability to quickly scan through large databases, find patterns that would have been otherwise hard to detect through other means, and use these patterns in order to accurately predict outcomes when given new sets of similar data (17). Thus, it becomes clear that ML has an increasing potential not only in the world of medical studies, but also in clinical practice. To the best of our knowledge, there is only one study that used ML in order to predict the probability of ISR occurrence by Jesús Sampedro-Gómez et al. (18).

In the present study, we used ML to build a prediction model which uses demographic, clinical and angiographic data in order to predict the appearance of ISR and the necessity of target lesion revascularization.

## Materials and methods

Patients with ACS who underwent PCI at Clinical Emergency Hospital, Bucharest, followed by a second coronary angiography, that occurred in the context of a new ACS, both between 2011 and 2019, were enrolled in this retrospective case-control study. We excluded patients whose data about the main outcome, ISR, could not be found. For the prediction of ISR we collected the following type of data: demographic: age and gender; cardiovascular risk factors: active smoking, smoke quitting compliance, dyslipidemia, HTN, DM; angiographic: extent of coronary artery disease (number of major coronary arteries with at least 70% stenosis, respectively 50% stenosis for lesions in the left main), location, length, diameter and type of stent, stent inflation pressure, the need for pre-dilation and/or post-dilation, presence of artery calcification and culprit lesion—whether the stented lesion was the one that cause the acute coronary syndrome(ACS) or not; other data: time passed between the PCI and the second angiography, whether the ACS was AMI or unstable angina, previous PCI or coronary artery by-pass graft (CABG). All the angiographic measurements were done by Quantitative Coronary Angiography in the core laboratory formed by interventional cardiologists with at least ten years of experience. The primary outcome evaluated was coronary in-stent restenosis, defined as a 50% or higher stenosis diameter in a coronary segment in which a stent had been previously implanted. The secondary outcome was the necessity of revascularization of the target lesion, defined as indication of PCI in a coronary segment with ISR. Addressing ISR severity, Mehran classification dividing ISR into four patterns was considered (19).

All analyses were completed using the statistical software program SPSS version 23. Data were presented as mean ± SD for continuous variables and as number and percentage for categorical counterparts. Differences between groups if normally distributed were compared with Student's *t*-test or chi-square, while for non-parametric variables Mann–Whitney *U*-test was assessed. *P*-values were two-tailed with a cut-off of less than 0.05 considered statistically significant.

We herein explored various ML techniques to predict the risk of ISR development within a certain timeframe, based on demographic, clinical parameters and risk factors. Overall, 21 features were selected as input variables for the ML algorithms, including continuous, categorical and binary variables. Therefore, in the preprocessing phase continuous features were rescaled to [0,1] and the categorical features have been one hot encoded.

**Figure 1:**
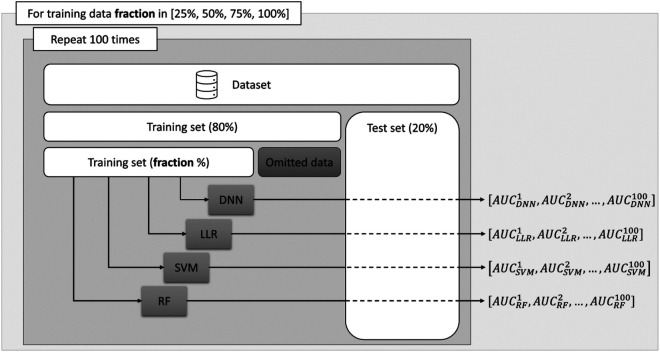
Schematic overview of the proposed analysis. The outer loop sets the fraction of data used and the inner loop randomly splits the dataset into a training and a testing set.

Experiment setup—given the relatively small number of available samples, the trained models may lack generalizability, and a regular performance evaluation on a held-out test set might not be representative. Therefore, as depicted in Figure 1 we propose a strategy to assess the performance of various ML algorithms as a function of the amount of data available for training.

First, for each experiment a random train-test split was performed designating 20% of the available samples as an evaluation set. Next, from the remaining 80% of the data allocated to the training set, we randomly select 25%, 50%, 75% and 100% of the samples to train a Random Forest (RF) model (20), a support vector machines (SVM) model (21), a simple linear logistic regression (LLR) and a deep neural network (DNN). To reduce potential biases stemming from the data, we repeated each experiment 100 times with different random splits and store the area under the receiver operating characteristic curve (AUC) for the binary classification problem. This allowed us to assess the performance of the ML algorithms more accurately, by computing the mean of the AUC scores obtained across the experiments, as well as to assess the performance trend with respect to the number of training examples.

Four different types of ML algorithms—as displayed in Figure 1 - were trained to predict ISR. Deep neural networks exhibit the greatest potential in modelling non-linear patterns in the data that could lead to a performant classifier. However, when provided with insufficient number of training samples they become difficult to train due to overfitting effects. Therefore, to assess the true modelling potential we concurrently employed 3 established machine learning algorithms that can be optimized on relatively fewer data-points as compared to DNNs.[PA(DERIAD11] The RF, SVM and LLR algorithms have been trained using the scikit-learn library (22) available in the Python programming language, using the default parameters.

The deep learning model has a fully connected architecture with three hidden layers of 64, 64 and 32 neurons respectively, where non-linearities are provided by hyperbolic tangent activation functions. The output layer is composed of only one neuron with a sigmoid activation, hence the model will output the probability for the input pertaining to the positive class. Given the reduced number of training examples, we regularized the model by a drop-out layer placed between the last hidden and the output, randomly removing 15% of the connections, thus forcing the model to perform predictions more objectively.

For the DNN model the fraction of data used for training was further randomly split into a train set (80% of the samples) and a validation set. The training was enabled by the Adam optimizer with an initial learning rate of 10^−4^, minimizing a binary cross-entropy cost function for 200 epochs, with a batch size of 64. Given the reduced number of training examples, for each experiment we chose the best performing model with respect to the validation loss to avoid the effect of overfitting. To count for the severe class imbalance (only 18% positive cases) we penalize the model more when misclassifying a positive sample, as compared to a negative one, by setting class weights computed using the scikit-learn library (22).

## Results

A number of 340 patients was admitted into the hospital for two diagnoses, namely unstable angina (37.20%) and acute myocardial infarction (62.80%). From this cohort, 72.76% were males with a mean age of 59.95 ± 11.07 years old, and 27.24% were females with a mean age of 64.93 ± 9.26 years. A total of 492 stents were implanted in these patients, out of which 158 (32.11%) were BMS, 18 (3.66%) were first-generation DES, 125 (25.41%) second-generation, and 132 (26.83%) third-generation DES. In-stent restenosis was found in 87 (17.68%) of the coronary lesions, out of which 42 (48.28%) required a second PCI with stent implantation. The demographic, clinical, angiographic and stent predictors for ISR considered into our study are displayed in Table 1.

**Table 1:** The clinical, angiographic, and stent predictors considered for in-stent restenosis probability.

Variables	All cohort *n* = 492	In-stent restenosis *n* = 87	Non In-stent restenosis *n* = 405	*p*-value	Chi-square
Age, years	61.31 ± 10.83	61.68 ± 10.23	61.23 ± 10.97	0.594	0.28
Gender, *n* (%)					
Female	134 (27.24)	28 (32.18)	106 (26.17)	0.390	0.74
Male	358 (72.76)	59 (67.82)	299 (73.83)		
Clinical predictors					
Reason for admission, *n* (%)					
UA	183 (37.20)	31 (35.63)	152 (37.53)	0.051	3.81
AMI	309 (62.80)	56 (64.37)	253 (62.47)		
Dyslipidemia, *n* (%)	371 (75.40)	63 (72.41)	308 (76.05)	0.560	0.34
Diabetes mellitus, *n* (%)	151 (30.69)	20 (22.99)	131 (32.35)	0.088	2.91
Arterial hypertension, *n* (%)	395 (80.28)	74 (85.06)	321 (79.26)	0.220	1.51
Smoking, *n* (%)	169 (34.35)	27 (31.03)	142 (35.06)	0.473	0.51
Smoking compliance, *n* (%)	108 (21.95)	18 (20.69)	90 (22.22)	0.621	0.24
Angiographic predictors					
Number of affected arteries, *n* (%)					
1	136 (27.64)	20 (22.99)	116 (28.64)	0.032	6.86
2	205 (41.67)	23 (26.44)	182 (44.94)		
3	151 (30.69)	44 (50.57)	107 (26.42)		
Stent location, *n* (%)					
RCA	187 (38.00)	42 (48.27)	145 (35.80)	0.108	7.59
LCx	74 (15.04)	8 (9.20)	66 (16.30)		
LM	17 (3.46)	3 (3.45)	14 (3.46)		
LAD	184 (37.40)	30 (34.48)	154 (38.02)		
Others	30 (6.10)	4 (4.60)	26 (6.42)		
Multiple stents, *n* (%)	267 (54.26)	39 (44.82)	228 (56.29)	0.172	1.67
Severity of stented lesion, *n* (%)					
Occlusion	141 (28.66)	30 (34.38)	111 (27.41)	0.183	1.78
95–90%	251 (51.02)	37 (42.53)	214 (52.84)		
80%	67 (13.62)	12 (13.79)	55 (13.58)		
75–70%	22 (4.47)	5 (5.75)	17 (4.20)		
<70%	11 (2.23)	3 (3.45)	8 (1.97)		
Culprit lesion, *n* (%)	423 (85.98)	79 (90.80)	344 (84.94)	0.157	2.00
Calcification, *n* (%)	80 (16.26)	11 (12.64)	69 (17.04)	0.316	1.01
Stent pre-dilatation, *n* (%)	332 (67.48)	64 (73.56)	268 (66.17)	0.533	0.35
Stent post-dilatation, *n* (%)	169 (34.35)	30 (34.48)	139 (34.32)	0.712	0.14
Stent predictors					
Stent length, *n* (%)					
2	1 (0.20)	0 (0.00)	1 (0.25)	0.606	0.27
8	18 (3.66)	6 (6.90)	12 (2.96)		
9	2 (0.41)	1 (1.15)	1 (0.25)		
10	3 (0.61)	1 (1.15)	2 (0.49)		
11	11 (2.24)	4 (4.60)	7 (1.73)		
12	19 (3.86)	3 (3.45)	16 (3.95)		
13	6 (1.22)	1 (1.15)	5 (1.23)		
14	27 (5.49)	3 (3.45)	24 (5.93)		
15	20 (4.07)	4 (4.60)	16 (3.95)		
16	16 (3.25)	2 (2.30)	14 (3.46)		
17	3 (0.61)	2 (2.30)	1 (0.25)		
18	71 (14.43)	7 (8.05)	64 (15.80)		
19	7 (1.42)	0 (0.00)	7 (1.73)		
20	24 (4.88)	1 (1.15)	23 (5.68)		
21	1 (0.20)	0 (0.00)	1 (0.25)		
22	6 (1.22)	2 (2.30)	4 (0.99)		
23	29 (5.89)	7 (8.05)	22 (5.43)		
24	59 (11.99)	8 (9.20)	51 (12.59)		
25	5 (1.02)	2 (2.30)	3 (0.74)		
26	3 (0.61)	0 (0.00)	3 (0.74)		
27	4 (1.22)	1 (1.15)	3 (0.74)		
28	55 (2.24)	7 (8.05)	48 (11.85)		
29	3 (0.61)	0 (0.00)	3 (0.74)		
30	6 (1.22)	1 (1.15)	5 (1.23)		
32	11 (2.24)	5 (5.75)	6 (1.48)		
33	26 (5.28)	8 (9.20)	18 (4.44)		
34	2 (0.41)	1 (1.15)	1 (0.25)		
36	9 (1.83)	1 (1.15)	8 (1.98)		
37	2 (0.41)	0 (0.00)	2 (0.49)		
38	29 (5.89)	6 (6.90)	23 (5.68)		
40	1 (0.20)	1 (1.15)	0 (0.00)		
48	13 (2.64)	3 (3.45)	10 (2.47)		
Stent diameter, *n* (%)					
2	3 (0.61)	1 (1.15)	2 (0.49)	0.014	6.03
2.25	13 (2.64)	2 (2.30)	11 (2.72)		
2.5	60 (12.20)	8 (9.20)	52 (12.84)		
2.7	1 (0.20)	0 (0.00)	1 (0.25)		
2.75	79 (16.06)	14 (16.09)	65 (16.05)		
3	152 (30.89)	30 (34.48)	122 (30.12)		
3.5	139 (28.25)	27 (31.03)	112 (27.65)		
4	36 (7.32)	4 (4.60)	32 (7.90)		
4.5	6 (1.22)	0 (0.00)	6 (1.48)		
5	2 (0.41)	1 (1.15)	1 (0.25)		
5.5	1 (0.20)	0 (0.00)	1 (0.25)		
Stent generation, *n* (%)					
BMS	158 (32.11)	54 (62.07)	104 (25.68)	<0.001	27.42
DES 1	18 (3.66)	4 (4.60)	14 (3.46)		
DES 2	125 (25.41)	12 (13.79)	113 (27.90)		
DES 3	132 (26.83)	11 (12.64)	121 (29.88)		
Maximum pressure, *n* (%)					
8	3 (0.61)	0 (0.00)	3 (0.74)	0.470	0.52
10	9 (1.83)	0 (0.00)	9 (2.22)		
11	1 (0.20)	0 (0.00)	1 (0.25)		
12	41 (8.33)	3 (3.45)	38 (9.38)		
13	5 (1.02)	0 (0.00)	5 (1.23)		
14	75 (15.24)	13 (14.94)	62 (15.31)		
15	11 (2.24)	2 (0.41)	9 (2.22)		
16	86 (17.48)	17 (4.20)	69 (79.31)		
17	1 (0.20)	0 (0.00)	1 (0.25)		
18	72 (14.63)	17 (4.20)	55 (13.58)		
20	18 (3.66)	4 (4.60)	14 (3.46)		
22	4 (0.81)	0 (0.00)	4 (0.99)		
24	2 (0.41)	0 (0.00)	2 (0.49)		
28	1 (0.20)	0 (0.00)	1 (0.25)		

AMI, acute myocardial infarction; BMS, bare metallic stents; DES, drug-eluting stents; LAD, left anterior descending artery; LCX, left circumflex; LM, left main coronary artery; UA, unstable angina.

Out of the stents, 17 (3.46%) were implanted on the left main (LM) coronary artery, 187 (38. 01%) on the RCA, 184 (37.40%) on LAD artery, 74 (15.04%) on LCX and 30 (6.10%) on other smaller branches or by-pass grafts. The average time spent between the two angiographies was 1.25 years. The characteristics of the implanted stents were also evaluated in terms of length, diameter, generation, and maximum pressure (Table 1). Concerning the angiographic Mehran classification of ISR cases, pattern I was found in 35% of patients, pattern II in 25%, pattern III in 31% and IV in 9%, *p* = 0.72.

Taking into account all the variables introduced in the model, the following regression equation was obtained:

(1) $$\begin{array}{rl} & \mathrm{In}\text{-}\mathrm{stent}\;\mathrm{restenosis}\;\mathrm{probability}\;=\;3.50\;+\;0.000043(\mathrm{TtF})\\ & -\;0.0262\;L_{\mathrm{St}}\;+\;0.0112\;St_{L}\;-\;1.163\;St_{D}\;+\;0.0558\\ & P_{\max}\;-\;0,0106\;A\;+\;0.0\;\mathrm{Dy}s_{\mathrm{yes}}\;+\;0.419\;\mathrm{Dy}s_{\mathrm{no}}\;+\;0.272\;\mathrm{Dy}s_{x}\;+\;0.0\;S_{\mathrm{yes}}\;-\;10\;S_{\mathrm{no}}\;-\;0.274\;\mathrm{Dia}b_{\mathrm{no}}\;-\;0.377\\ & \mathrm{AH}T_{\mathrm{no}}\;+\;0.0\;\mathrm{Ang}\;+\;0.872\;\mathrm{AMI}\;+\;1.153\;No_{1}\;+\;0.0\;No_{2}\;+\;0.310\;No_{3}\;+\;0.0\;\mathrm{Lo}c_{\mathrm{RCA}}\;-\;2.136\;\mathrm{Lo}c_{\mathrm{CA}}\;-\;0.474\\ & \mathrm{Lo}c_{\mathrm{AIVA}}0.77\;\mathrm{Lo}c_{\mathrm{CT}}\;-\;1.65\;\mathrm{Lo}c_{O}\;+\;0.0\;\mathrm{Cu}l_{\mathrm{yes}}\;\;+\;1.097\;\mathrm{Cu}l_{\mathrm{no}}\;+\;0.0\;\mathrm{Ge}n_{0}\;+\;0.38\;\mathrm{Ge}n_{1}\;-\;1.877\;\mathrm{Ge}n_{2}\;-\;2.638\\ & \mathrm{Ge}n_{3}\;-\;2.28\;\mathrm{Ge}n_{x}\;+\;0.0\;\mathrm{Pre}D_{\mathrm{yes}}\;-\;0.356\;\mathrm{Pre}D_{\mathrm{no}}\;+\;0.0\;\mathrm{Post}D_{\mathrm{yes}}\;-\;0.226\;\mathrm{Post}D_{\mathrm{no}}\;+\;0.555\;C_{\mathrm{no}}\;+\;0.0\\ & SC_{\mathrm{yes}}\;+\;10\;SC_{x}\;-\;0.088\;SC_{\mathrm{no}}\;+\;0.0\;At_{\mathrm{yes}}\;-\;0.416\;At_{\mathrm{no}}\;+\;0.0F\;-\;0.360\;M \end{array}$$

Where TtF–time to follow-up

L_St_–stented lesion, %

St_L_–stent length

St_D_–stent diameter

P_max_–maximum pressure

A–age

Dys_yes_–with dyslipidemia

Dys_x_–unknown dyslipidemia status

S_yes_–smoking

S_no_–non-smoking

Diab_yes_–with diabetes

AHT_yes_–with arterial hypertension

Ang–hospitalized for unstable angina

AMI–hospitalized for acute myocardial infraction

No_1_–one affected artery

No_2_–two affected arteries

No_3_–three affected arteries

Loc_RCA_–stent located in the right coronary artery

Loc_CA_–stent located in the circumflex artery

Loc_AIVA_–stent located in the anterior interventricular artery

Loc_CT_–stent located in the coronary trunk

Loc_O_–stent located in other vessels

Cul_yes_–with culprit lesion

Cul_no_–without culprit lesion

Gen_0_–stent generation 0

Gen_1_–stent generation 1

Gen_2_–stent generation 2

Gen_3_–stent generation 3

Gen_x_–stent generation unkonown

PreD_yes_–with predilatation

PreD_no_–without predialtation

PostD_yes_–with postdilatation

PostD_no_–without postdilatation

C_yes_–with calcification

C_no_–without calcification

SC_yes_–with smoking compliance

SC_x_–smoking compliance unknown

SC_no_–with smoking compliance

At_yes_–with atheromatosis history

At_no_–without atheromatosis history

F–female

M–male

Simplifying Equation 1 by removing the terms with null coefficient, we obtain the following relation:

(2) $$\begin{array}{rl} & \mathrm{In}\text{-}\mathrm{stent}\;\mathrm{restenosis}\;\mathrm{probability}\;=\;3.50\;+\;0.000043\;\mathrm{TtF}-\;0.0262\;L_{\mathrm{St}}\\ & +\;0.0112\;St_{L}\;-\;1.163\;St_{D}\;+\;0.0558\\ & P_{\max}\;-\;0,0106\;A\;+\;0.419\;\mathrm{Dy}s_{\mathrm{no}}\;+\;0.272\;\mathrm{Dy}s_{x}\;-\;10\;S_{\mathrm{no}}\;-\;0.274\;\mathrm{Dia}b_{\mathrm{no}}\;-\;0.377\;\mathrm{AH}T_{\mathrm{no}}\;+\;0.872\;\mathrm{AMI}\;+\;1.153\\ & No_{1}\;+\;0.310\;No_{3}\;-\;2.136\;\mathrm{Lo}c_{\mathrm{CA}}\;-\;0.474\;\mathrm{Lo}c_{\mathrm{AIVA}}0.77\;\mathrm{Lo}c_{\mathrm{CT}}\;-\;1.65\;\mathrm{Lo}c_{O}\;+\;1.097\;\mathrm{Cu}l_{\mathrm{no}}\;+\;0.38\\ & \mathrm{Ge}n_{1}\;-\;1.877\;\mathrm{Ge}n_{2}\;-\;2.638\;\mathrm{Ge}n_{3}\;-\;2.28\;\mathrm{Ge}n_{x}\;+\;0.0\;\mathrm{Pre}D_{\mathrm{yes}}\;-\;0.356\;\mathrm{Pre}D_{\mathrm{no}}\;-\;0.226\;\mathrm{Post}D_{\mathrm{no}}\;+\;0.555\\ & C_{\mathrm{no}}\;+\;10\;SC_{x}\;-\;0.088\;SC_{\mathrm{no}}\;-\;0.416\;At_{\mathrm{no}}\;+\;0.0F\;-\;0.360\;M \end{array}$$

From this set of predictors only 3 predictors were statistically significant (*p* < 0.05) at a confidence interval of 95%, precisely, the number of affected arteries, stent generation and diameter.

Corroborating the information from Table 1 and Equation 2, it can be concluded that stents from older generations increase the risk of in-stent restenosis, while generations 2 and 3 diminish the probability of this condition. In our study, 62.07% of in-stent restenosis occurred in patients with BMS, whereas generations 2 and 3 generated 13.79%, and respectively, 12.64% of the restenosis cases. Furthermore, reduced diameter of the stent, precisely less than 3.5 mm related with the risk of ISR.

**Figure 2:**
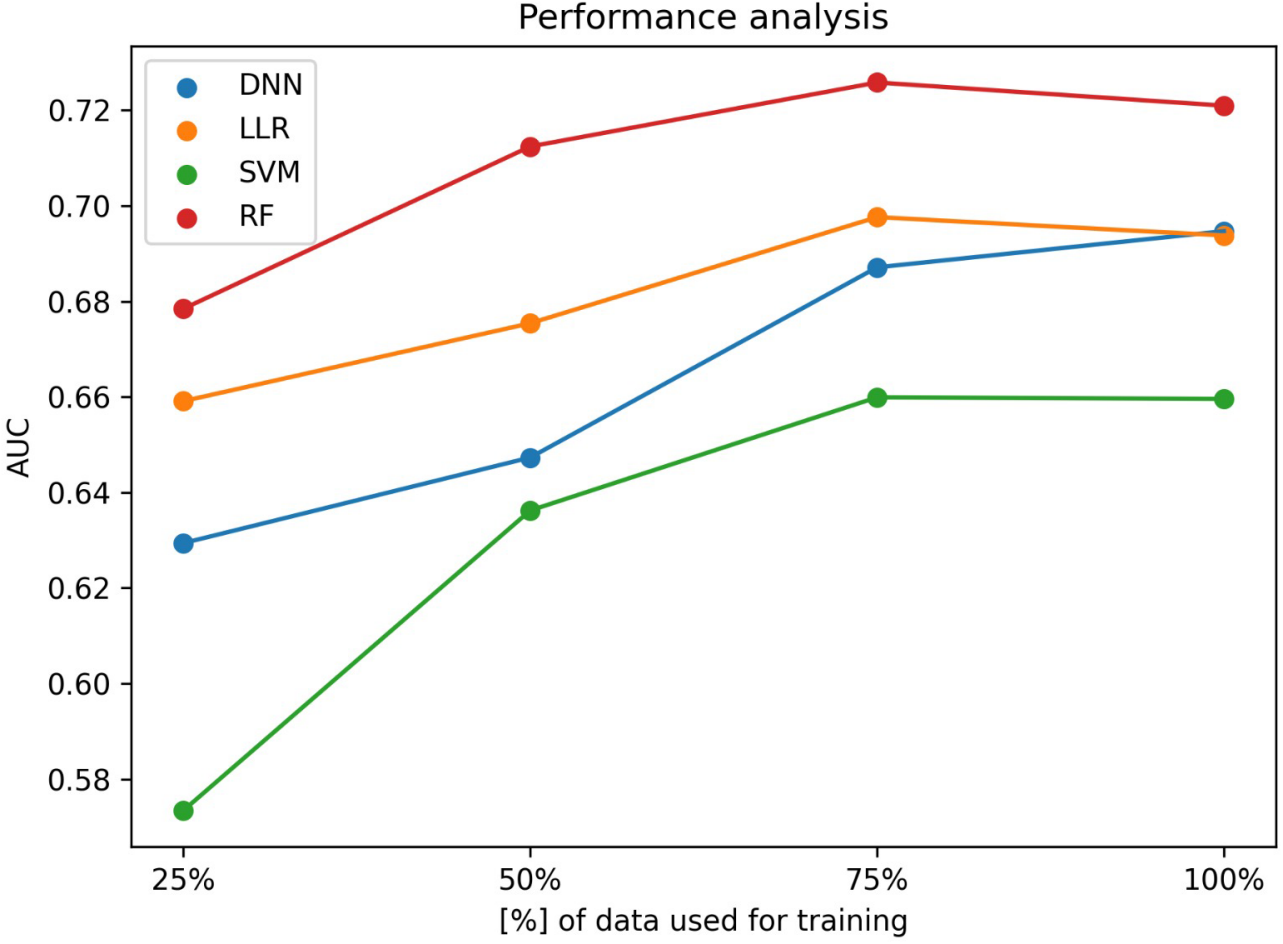
Machine learning performance as a function of the dataset size. y-axis shows the ROC-AUC while the x-axis shows the fraction of data used for training a DNN (blue), a LLR (orange), a SVM (green) and a RF (red) prediction model.

Concerning the ML results, as described in the subsection Experiment setup we aimed to assess the effect of increasing the number of training samples on the model performance. Figure 2 shows the mean AUCs for each experiment.

All algorithms have shown a statistically significant (*p*-value < 0.05) improvement in AUC when increasing the data fraction from 25% to 100%, but none of them has reached a statistically significant improvement when increasing the fraction of data from 75% to 100%.

As depicted in Table 2, when using the entire training set to train the models, in terms of AUC scores, the best performing algorithm is the random forest, achieving a mean AUC of 0.721 [95% CI (0.709–0.733)]. In terms of the algorithm performance trend, although not statistically significant (*p*-value = 0.47), the DNN is the only one that seems to maintain an ascending trendline when increasing the fraction of data used for optimization from 75% to 100%, while all other algorithms seem to saturate. When assessing the impact of increasing the fraction used for training from 50% to 100%, random forest algorithm still shows no statistically significant improvement (*p*-value = 0.32) while the DNN does (*p*-value = 3.87 × 10^−5^).

**Table 2:** Machine learning experiment area under the curve (AUC) score results.

Fraction of data used for training	DNN	SVM	LLR	RF
25%	0.629	0.573	0.659	0.678
50%	0.647	0.636	0.675	0.712
75%	0.687	0.660	0.698	0.726
100%	0.695	0.660	0.694	0.721

DNN, deep neural network; LLR, linear logistic regression; RF, forest; SVM, support vector machines.

## Discussions

The implementation of specific recommendations in a personalized approach is a desirable goal for any disease. Machine learning algorithms aiming to predict high vs. low risk subjects for ISR could avoid unnecessary follow-ups and consequently costs.

The primary aim of the study was to investigate the risk factors for in-stent restenosis considering multiple clinical and angiographic variables from both a classic statistical perspective, but also from a ML approach.

The statistical analysis pinpointed three elements increasing the risk for ISR: stent generation and diameter, as well as the number of affected arteries, subjects with multiple coronary lesions being at a higher risk. Similarly to findings from literature (23), the majority of ISR cases were registered in subjects with BMS, compared with second and third generation of DES. Moreover, ISR was more frequently in small diameter stents and in multi-vessel coronary disease patients; 94.25% of the total individuals with ISR had stents with diameters between 2 and 3.5 mm in agreement with the other reports (9). Compared with previous studies that revealed a relation between ISR and other risk factors, such as smoking, DM, HTN, dyslipidemia, impaired renal function, etc., this was not identified in our cohort (24). Referring to the angiographic morphologic classification of ISR, 60% of our cases were Mehran pattern I or II. Besides the Mehran classification applied in the present study that has some inconvenient as it was designed in the BMS area, there are other methods of ISR classification, such as the use of intracoronary imaging, such as intravascular ultrasound (IVUS) or optical coherence tomography (OCT), Waksman classification (25), that allows the determination of the underlying mechanisms involved in the development of ISR, for guidance and optimization of the results. Unfortunately, in the current study only a minority of patients (around 20%) beneficiated of intracoronary imaging, reason for which this predictor could not be included in our model.

Starting from the same cohort we have built four ML algorithms to predict ISR, from which RF showed the best areas under the curve with increasing fraction of data used for training, but even if DNN seem to perform worse on small datasets as compared to simpler ML algorithms such as LLR or RF, it has greater potential in achieving a better performance when the number of samples available for training increases. Compared with the only study that utilized ML to predict restenosis (18), our models have external validation at least for the subjects that presented with another ACS and the RF algorithm from the present research displayed better AUCs compared with GRACIA-3, EVENT or PRESTO studies. It is to emphasize that ML models work properly when extensive balanced data are provided. Nevertheless our models included small, unbalanced data sets that reflect better the daily clinical scenarios, but impacting the generality of the model. Furthermore it is also important to compare ML algorithms with conventional methods of ISR risk assessment, such as coronary angiography or intracoronary imaging (optical coherence tomography-OCT and intravascular ultrasound-IVUS). Proper stent expansion is a well-known factor for preventing ISR and several studies showed that smaller minimal stent area at IVUS are relative good predictor for ISR (26) (AUC varying around 0.8 depending on the coronary artery location) mainly in left main stenting, but less studied for other locations, reason for which it is a 2a recommendation only for left main and complex coronary artery stenting in the 2021 American Heart Association/American College of Cardiology, and Society of Cardiovascular Angiography and Interventions (AHA/ACC/SCAI) Guideline for Coronary Artery Revascularization. Even if intracoronary imaging has many advantages in predicting ISR its use is recommended for optimizing stenting mainly in the left main and its wide application restricted due to increased costs. The proposed ML model combining of clinical and angiographic characteristics offers clinicians a tool for early identification of high-risk patients for ISR allowing clinicians to early detect risk factors.

## Limitations

Firstly, our study is limited by its retrospective, single-center design. The indication for repeating the coronary angiography was based on new ischemic events, therefore the patients included could represent a selected high-risk group questioning the validation of our cohort. Secondly, there are other predictors, such as intravascular imaging, biomarkers, genetic elements that were not included into our model. Thirdly, there are patients with asymptomatic ISR that have not been included into our study, bias inducing an underestimation of the accuracy of the displayed model.

## Conclusions

Using routine clinical and angiographic data in a retrospective analysis we have built 4 machine learning models to predict the risk factors of ISR with good prediction scores for RF model that could help clinicians in deciding coronary stenting.

## Data Availability

The raw data supporting the conclusions of this article will be made available by the authors, without undue reservation.
